# An upstream approach to veterinary workforce wellbeing: an evaluative review of an embedded wellbeing curriculum in veterinary education

**DOI:** 10.3389/fvets.2026.1972718

**Published:** 2026-09-15

**Authors:** Virginia K. Corrigan, Jennifer Carter, Rachel H. Colla, Laura Dooley, Lara H. Mossman, Dianne A. Vella-Brodrick

**Affiliations:** 1 University of Tennessee College of Veterinary Medicine, Knoxville, TN, United States; 2 Melbourne Veterinary School, Faculty of Science, The University of Melbourne, Parkville, VIC, Australia; 3 Centre for Wellbeing Science, Faculty of Education, The University of Melbourne, Parkville, VIC, Australia

**Keywords:** competency-based education, curriculum evaluation, learning design, veterinary education, wellbeing

## Abstract

Veterinary professionals work in demanding environments shaped by individual, relational, organizational, and systemic pressures. Veterinary education can therefore serve as an upstream setting for developing both clinical competence and the capabilities needed for sustainable professional engagement and wellbeing. Competency-based veterinary education offers a structure for treating wellbeing as a professional capability that can be taught, practiced, assessed, and evaluated. This study reports an evaluative review of a longitudinal wellbeing curriculum embedded within the Doctor of Veterinary Medicine program at the Melbourne Veterinary School. Data sources included curriculum documents, curriculum mapping, two non-participant teaching observations, and collaborative interpretation by veterinary education and wellbeing science experts. AAVMC CBVE® 2.0 and the Wellbeing Integrated Learning Design framework served as evaluative lenses. The review examined how wellbeing science was integrated across the formal curriculum and identified priorities for strengthening alignment, delivery, assessment, and evaluation. The intended learning outcomes were conceptually appropriate, developmentally sequenced, and aligned most strongly with CBVE® Domain 7. However, constructive alignment with assessment was incomplete. Opportunities for applied practice and feedback were limited, no explicit formal wellbeing curriculum was present in the final clinical year, and links among wellbeing, communication, and systems-level contributors were infrequent. Priorities included greater vertical integration, intentional learning design, applied professional practice, formative reflection, educator development, and longitudinal evaluation. Because systematic student and educator feedback was not collected, the findings describe the formal curriculum but do not establish how it was experienced or whether it improved learner outcomes. The study offers an adaptable process for reviewing, refining, and evaluating embedded wellbeing curricula within existing veterinary education structures from a systems-informed perspective.

## 1 Introduction

Veterinary medicine continues to face substantial challenges related to burnout, mental ill health, suicide risk, moral distress, workforce retention, and access to care (1–8). These workforce challenges also intersect with the broader societal responsibilities of the veterinary profession. The One Health High-Level Expert Panel defined One Health as an integrated, unifying approach intended to sustainably balance and optimize the health of people, animals, and ecosystems, recognizing their interdependence and the need for collaboration across disciplines and sectors (9). Veterinary professionals contribute to this agenda through animal health and welfare, zoonotic disease prevention, food safety and security, antimicrobial stewardship, public health, environmental stewardship, and cross-sector collaboration (10).

Sustaining these contributions requires both clinical and technical competence and a veterinary workforce capable of functioning effectively within complex systems over time. Veterinary education may therefore be understood as an upstream and enabling contribution to One Health and to Sustainable Development Goal 3, Good Health and Wellbeing, and Sustainable Development Goal 4, Quality Education (11). Preparing future veterinary professionals extends beyond clinical and technical day-one competence to include interpersonal skills, intrapersonal skills, and an understanding of the complex systems in which veterinary care is delivered (12). Higher education can help future veterinary professionals develop the knowledge and capacities to navigate and influence these systems, supporting personal and professional wellbeing, care provision, and career sustainability (12).

Historically, many wellbeing initiatives in both higher education and veterinary education have focused on supporting individuals through optional services, stress management, resilience training, or self-care interventions. While these supports remain important, a growing body of wellbeing science and higher education scholarship suggests that wellbeing should also be understood as a core feature of teaching, learning, and institutional culture rather than as an adjunct or “add-on” support service (13–15). This shift reframes wellbeing from something individuals are expected to manage toward something that can be intentionally supported through curriculum design, learning activities, assessment practices, educator development, and the broader learning environment (16, 17).

Several complementary conceptualizations of wellbeing inform this shift. The dual-continua model distinguishes mental health from mental illness, emphasizing that the absence of distress does not necessarily indicate the presence of flourishing (18). Multidimensional models of flourishing further highlight domains such as meaning, purpose, relationships, and physical and mental health (19). Recent scholarly work re-defines flourishing in terms of “sustainable wellbeing,” involving balance and harmony across people, systems, environments, and time (20). These perspectives converge in a global educational paradigm shift toward recognizing flourishing as a central aim of education. For example, UNESCO has proclaimed that education should be reoriented toward human flourishing (21, 22). An international consensus paper also argues that flourishing provides an ethically defensible and empirically supported aim for educational systems (13).

A capability-oriented view of wellbeing, emphasizing its capacity to be developed through training, is particularly relevant to veterinary and other health professional education. Dahl et al. (23) propose four capabilities that support human flourishing: awareness, connection, insight, and purpose. These capabilities can be developed through active training to enable both wellbeing and deeper learning (23). A theory of public wellbeing extends this idea by emphasizing that wellbeing abilities are socially developed and contextually exercised through interactions between individuals and their environments (24). Wellbeing literacy similarly positions wellbeing as the capability to understand, communicate, and intentionally compose wellbeing-related language and actions across contexts (25, 26). Together, these frameworks suggest that wellbeing capacities can be developed in learners through intentional educational design.

Capability development is shaped by the environments in which students study, work, and relate to others. Self-Determination Theory (SDT), and particularly Basic Psychological Needs Theory, provide important wellbeing science mechanisms for understanding these person-environment interactions. SDT proposes that wellbeing, motivation, and learning are supported when the basic psychological needs of autonomy, competence, and relatedness are satisfied, and undermined when these needs are frustrated (27–30). The tenets of SDT therefore provide an important basis for curriculum design intended to foster flourishing in educational contexts (29). In veterinary education; curriculum structures, learning activities, assessment systems, feedback cultures, and faculty-student relationships can either support or create barriers to student wellbeing and learning. Recent veterinary education research supports this view. In a survey of 202 veterinary medical students across the United States, McDavid and San Miguel (31) found that perceptions of institutional social climate predicted psychological need satisfaction and frustration, which in turn predicted hope, life satisfaction, and burnout. Their findings highlight the central role of relationships in creating learning environments that support student thriving (31).

Students and veterinary professionals as individuals should not be expected to simply adapt to unhealthy systems. Contemporary veterinary wellbeing scholarship increasingly calls for a “both/and” approach: building individual capabilities, while also addressing structural and cultural contributors to distress (12). Cordova et al. (12) explicitly reject the false dichotomy between “fix the system” and “teach resilience,” proposing instead a staged, developmental, career-long model of veterinary wellbeing training that begins early in veterinary school, continues through clinical training, and extends into postgraduate professional development. This contemporary model organizes wellbeing capabilities into intrapersonal, interpersonal, and systems/sustainability domains, providing a veterinary-specific framework for a longitudinal and developmentally sequenced wellbeing education. The World Small Animal Veterinary Association (WSAVA) professional wellness guidelines correspondingly articulate wellbeing as a shared responsibility across individual, workplace, and professional levels (32).

The Wellbeing Integrated Learning Design (WILD) framework offers a practical pedagogical approach for translating these ideas into curriculum and teaching practice (16, 33). WILD draws on wellbeing and learning science to position wellbeing as a property of effective learning environments rather than as an additional content area (16). The framework, which is grounded in SDT, invites educators to consider how elements such as the wellbeing of the educator, learning environments, and assessment design shape the conditions under which both wellbeing and learning can emerge together. WILD was developed and initially trialed in Australian tertiary education rather than specifically in veterinary education (16, 33). WILD may be a feasible option for veterinary professional education because it provides a way to embed wellbeing science into existing teaching and learning approaches without adding new content to an already crowded curriculum.

The American Association of Veterinary Medical Colleges (AAVMC) Competency-Based Veterinary Education model (CBVE®) provides the existing structure for making wellbeing capacities visible as day-one professional competencies. CBVE® emphasizes observable, assessable professional competencies and constructive alignment among intended learning outcomes, learning activities, and assessment tasks [AAVMC, 2024; (34–36)]. CBVE® 2.0 was developed as a competency framework for veterinary education programs. Its core domains and competencies are intended to provide a common structure, implementation and subcompetencies may be adapted to local mission, context, culture, and accreditation requirements (37). Wellbeing is currently represented in the CBVE® 2.0 framework explicitly within Domain 7, Professionalism and Professional Identity. Competency 7.5, “attends to wellbeing of self and others,” infers that students will be able to recognize signs of stress in themselves and their colleagues, to engage in self-care and to recognize when professional support is appropriate, to manage the expectations of clients and of themselves, and to recognize sources of workplace stress and act to remedy adverse situations. Related competencies address reflection on personal actions and the use of feedback to plan improvement (7.3), self-directed learning (7.4), and career planning (7.6). These competencies are framed largely as individual competencies.

Wellbeing in veterinary education is maturing and evolving, and yet important gaps remain. Existing literature describes a range of curricular and co-curricular wellbeing interventions, practices, activities, and student support initiatives in veterinary education programs (2, 38–40). The available evidence has often focused on discrete or short-term interventions, with less attention to how wellbeing is being embedded longitudinally within required coursework, aligned with competency frameworks, enacted through pedagogy, assessed, and refined over time within real-life institutional constraints (39). There is also limited practical guidance for veterinary educators seeking to review and strengthen existing wellbeing curriculum without creating parallel programs or adding to already unsustainable workloads.

Curriculum review and refinement are therefore important tools and processes for the ongoing evolution of wellbeing in veterinary education. As wellbeing science, CBVE®, and contemporary educational practice continue to develop and evolve, veterinary programs need structured processes for examining how wellbeing-related competencies, teaching practices, assessment tasks, and learning environments align with one another and with the current and future needs of veterinary students, educators, stakeholders, and the profession. Translating wellbeing science into everyday educational practice and systems requires a paradigm shift in the current mental model from teaching *about* wellbeing to teaching *for* wellbeing, which implies structural integration rather than additive curricular content or programming (14, 16).

This article presents an evaluative curriculum review of a longitudinal, explicit wellbeing curriculum stream within the Doctor of Veterinary Medicine (DVM) program at Melbourne Veterinary School. This evaluation was undertaken to inform a planned program-wide curriculum review, with a specific focus on the wellbeing stream. The review applied the WILD and AAVMC CBVE® 2.0 frameworks to evaluate how wellbeing is integrated across the curriculum and identify priorities for revising this learning stream during the forthcoming program-wide curriculum review. Specifically, the paper considers the alignment of learning outcomes with CBVE® and the design of teaching resources and learning activities. It further explores priorities for future development across the DVM program, opportunities for formative and longitudinal assessment, and approaches for evaluating student learning and adapting teaching practices and the learning environment over time. In doing so, this paper offers a practical example of how veterinary schools may review, refine, and sustain wellbeing science within existing program curriculum and structures, in ways that connect veterinary professional wellbeing to broader One Health and global education priorities.

This evaluative review project arose from discussions between Melbourne Veterinary School faculty responsible for DVM curriculum oversight and the design and delivery of the wellbeing stream and a visiting veterinary educator and wellbeing researcher. The review was undertaken to inform a forthcoming program-wide curriculum review, with particular attention to the wellbeing stream. The curriculum had been developed over a period of ten years, with the version reported here having been delivered during the five years preceding the study. The review team collaboratively developed the following questions based on priorities identified through these discussions:

1. How well do the current wellbeing-related intended learning outcomes reflect the capabilities expected of veterinary graduates and align with AAVMC CBVE® 2.0?
2. How well do the existing teaching resources, learning activities, and session designs support achievement of these intended learning outcomes?
3. What gaps exist in the content and sequencing of the explicit wellbeing stream, and which areas should be prioritized for future development, particularly in the third year (DVM3) and fourth year (DVM4) of the curriculum?
4. Which assessment tasks could be introduced or refined to strengthen alignment with the wellbeing-related intended learning outcomes?
5. How could student learning and the learning environment be evaluated longitudinally to guide ongoing curriculum refinement?

Although the five questions focus on the DVM program’s wellbeing stream, the review applied a broader, systems-informed lens. Consistent with the shift towards wellbeing education that extends beyond discrete content to embedded, systems-informed, competency-based approaches, the review considered how learning design, pedagogy, assessment, and program-level conditions shape capacity development and support wellbeing-related competencies. The questions and systems lens together organized the analysis across five interrelated areas: intended learning outcomes, teaching and learning design, curricular content and sequencing, assessment alignment, and longitudinal evaluation.

## 2 Materials and methods

### 2.1 Study design

This study employed an evaluative curriculum review design integrating qualitative document analysis, curriculum mapping, observations of teaching and the learning environment, and collaborative interpretation with experts in wellbeing science and education. Consistent with established practice in health professions education, curriculum mapping was used to make relationships among intended outcomes, content, pedagogy, and assessment visible (41). Constructive alignment guided examination of coherence among intended learning outcomes, learning activities, and assessment tasks (34). Conducted as part of a U. S. Fulbright Scholar supported professional project on systems-informed wellbeing science and wellbeing-integrated learning design in veterinary education, the review adopted an interpretive, developmental orientation consistent with formative peer review of teaching. Its purpose was to identify strengths, gaps, and feasible priorities for revision and refinement using evidence and theory from wellbeing and learning science, including how to inform future program evaluation.

### 2.2 Setting and case context

The Melbourne DVM program includes a longitudinal wellbeing stream across the first 3 years of the 4-year DVM curriculum. Wellbeing content is embedded within required professional practice subjects: Introduction to Veterinary Practice (IVP) in the first year of the curriculum (DVM1), and Veterinary Professional Practice (VPP) 1 to 4 in the second year (DVM2) and third year (DVM3). Existing content includes professional identity, wellbeing, resilience, mindfulness and self-compassion, character strengths, sustainable engagement, burnout prevention, and emotional intelligence. At the time of review, there was no explicit formal wellbeing curriculum in DVM4, although existing staff mentor structures beginning in DVM3 and continuing through DVM4 provided an important foundation for potential clinical-year integration of wellbeing. Table 1 summarizes the year of delivery, format, and scheduled contact time for each wellbeing topic.

**Table 1:** Overview of the Melbourne Veterinary School DVM wellbeing curriculum (2026).

Year	Wellbeing topic	Format	Contact time
DVM1	Professional identity	2 h large-group workshop + 30 min online pre-work	2.5 h
DVM1	Wellbeing and resilience	2 × 1 h small-group tutorials + 1 h online video	3 h
DVM2	Mindfulness and self-compassion	1 h large-group seminar + 1.5 h small-group tutorial	2.5 h
DVM2	Character strengths	1 h online video + 1 h small-group tutorial	2 h
DVM3	Sustainable engagement / burnout prevention	1 h lecture + 1.5 h tutorial	2.5 h
DVM3	Emotional intelligence	1 h seminar	1 h
DVM4	No explicit formal wellbeing curriculum; staff mentor program in place	Mentor meetings attempted approximately every 8–10 weeks, constrained by clinical scheduling and workload	Variable

DVM = Doctor of Veterinary Medicine. Wellbeing content in DVM1 is delivered within Introduction to Veterinary Practice (IVP); content in DVM2 and DVM3 is delivered within Veterinary Professional Practice (VPP) 1–4.

Across the first 3 years, dedicated wellbeing content accounts for approximately 13.5 h of scheduled contact time, delivered through a blend of online pre-work, large-group sessions, and small-group tutorials.

### 2.3 Data sources

Data sources included existing curriculum materials from the Melbourne Veterinary School DVM wellbeing curriculum, including intended learning outcomes, progress expectations, topic outlines, teaching materials, facilitator resources, and available curriculum mapping documents. VKC also conducted two non-participant observations of selected teaching and learning contexts: a DVM1 session at Parkville on 31 March 2026 and a DVM2 session at Werribee on 1 April 2026. Observation notes were used to provide contextual insight into pedagogy, educator–student interactions, the learning environment, and opportunities for participation and reflection. Additional contextual sources included documented collaborative feedback from Melbourne Veterinary School and Centre for Wellbeing Science partners. Curriculum materials reviewed were those in use during the 2026 academic year. Document analysis was conducted as a qualitative research method (42).

### 2.4 Analytic lenses

Two analytic lenses guided the review. The first was CBVE® 2.0, with emphasis on Domains 5, 6, and 7: Communication; Collaboration; and Professionalism and Professional Identity (35, 36). The WILD framework was used as the primary lens for considering how wellbeing could be embedded through learning design, teaching practice, learning environment, and assessment (16, 33). WILD was applied as an external evaluative lens, in a manner analogous to a structured peer review of teaching tool, to describe which wellbeing-supportive design features were already present in current practice and where opportunities existed to strengthen them.

### 2.5 Analytic process

The analysis used a primarily deductive, framework-guided qualitative document analysis combined with curriculum mapping and structured interpretive synthesis. The five educator-led questions served as *a priori* organizing domains, and the two analytic lenses described above provided the criteria through which the curriculum materials and observation notes were examined. Evidence was organized in working matrices and crosswalks to compare intended learning outcomes, content, sequencing, learning activities, pedagogical and learning-environment features, assessment practices, and opportunities for longitudinal evaluation.

VKC served as the primary evaluator and conducted the initial review of the source materials, mapped the intended learning outcomes and progress expectations to CBVE® 2.0, applied the WILD framework to the teaching resources and learning design, and integrated the observation notes with the documentary findings. This initial analysis generated provisional mappings and evaluations concerning curriculum strengths, gaps, alignment, development priorities, assessment opportunities, and evaluation strategies.

The provisional analysis was then reviewed iteratively by the author team. LD and JC contributed contextual knowledge of the Melbourne DVM program, curriculum oversight, and the feasibility of the proposed recommendations. LD was a designer of the original curriculum, in collaboration with discipline experts. RHC, LHM, and DAVB examined the analysis and recommendations through wellbeing science, WILD, and systems-informed perspectives. Their review was used to test the conceptual coherence, contextual accuracy, evidence-based approach, and practical feasibility of the interpretations.

The authors did not independently conduct duplicate analyses of the full dataset. Instead, agreement was reached through successive rounds of collaborative review and discussion. Where interpretations differed, the team returned to the relevant curriculum materials, observation notes, research questions, and framework definitions and revised the analysis until consensus was reached. Because the purpose was formative, interpretive curriculum evaluation rather than independent categorical coding, inter-rater reliability statistics were not calculated. The agreed synthesis informed an internal curriculum review report and was subsequently refined for this manuscript, with attention to the feasibility of recommendations within constraints related to timetabling, clinical workload, educator capacity, assessment burden, and existing program structures.

## 3 Results

The findings reported below concern the formal and documented curriculum and the review team’s framework-guided interpretation of those materials. They do not represent systematic evidence of how students or educators experienced the curriculum or whether the curriculum produced changes in wellbeing-related capabilities or outcomes.

### 3.1 Research question 1: appropriateness of intended learning outcomes and alignment with CBVE®

The current intended learning outcomes appear to be appropriate for a multi-year, sequenced wellbeing curriculum and show conceptual alignment with CBVE®, particularly Domain 7 (professionalism and professional identity). The strongest alignments are with reflection, self-directed learning, career planning, and attending to the wellbeing of self and others. Secondary alignments are visible across Domains 5 (communication) and 6 (collaboration).

Table 2 presents the full crosswalk produced by this mapping. A crosswalk in this context is a point-by-point correspondence table: each wellbeing topic, together with its intended learning outcomes and progress expectations, is set against the CBVE® 2.0 competencies it most directly supports (primary alignment) and those it supports more indirectly (secondary alignment).

**Table 2:** Crosswalk of the Melbourne DVM wellbeing curriculum and intended learning outcomes with CBVE® 2.0 competencies.

DVM year / course	Wellbeing topic	Intended learning outcomes and progress expectations	CBVE® primary alignment	CBVE® secondary alignment
DVM1 IVP	Introduction to wellbeing	Intended learning outcomes Define wellbeing. Explain the factors that influence wellbeing, both positively and negatively. Progress Expectations Reflect on past experiences and current choices that influence personal wellbeing. Evaluate the challenges and barriers to maintaining wellbeing. • Apply an understanding of strategies and interventions that support wellbeing to create a personalized wellbeing plan.	7.3 Reflects on personal actions and uses feedback to plan improvement 7.5 Attends to wellbeing of self and others	7.2 Practices time management 7.4 Engages in self-directed learning
DVM1 IVP	Resilience	Intended learning outcomes Define resilience. Explain the factors that contribute to resilience. Identify adaptive strategies to build resilience. Progress expectations Reflect on personal resilience, including individual triggers and signs of stress. Identify adaptive coping strategies to build personal resilience. Formulate a personalized resilience plan.	7.5 Attends to wellbeing of self and others	7.2 Practices time management 7.3 Reflects on personal actions and uses feedback to plan improvement
DVM1 IVP	Introduction to professional identity	Intended learning outcomes Explain the concept of veterinary professional identity, and describe how personal, cultural, and workplace factors shape veterinarians’ values and decision-making, using examples. Reflect on personal motivations for studying veterinary medicine and how these shape career aspirations. List multiple dimensions of diversity relevant to veterinary workplaces and evaluate at least two ways that diverse perspectives can improve clinical reasoning and team functioning in veterinary contexts.	7.1 Adopts an ethical approach to meeting professional obligations 7.3 Reflects on personal actions and uses feedback to plan improvement	6.1 Solicits, respects and integrates contributions from others 6.4 Demonstrates inclusivity and cultural competence 7.6 Engages in career planning
DVM2 VPP1	Mindfulness and self-compassion	Intended learning outcomes Describe the difference between wellbeing and success. Define mindfulness and self-compassion and describe their impact on personal wellbeing. Explain the value of developing greater mindfulness and self-compassion as a veterinarian. Describe strategies that develop and sustain the psychological resources of mindfulness and self-compassion.	7.3 Reflects on personal actions and uses feedback to plan improvement 7.5 Attends to wellbeing of self and others	7.4 Engages in self-directed learning
DVM2 VPP2	Character Strengths	Intended Learning Outcomes Define character strengths and describe their impact on personal wellbeing. Explain the value of developing your character strengths as a veterinarian. Describe strategies that develop character strengths. Progress expectations Demonstrate self-awareness of individual character strengths. • Apply an understanding of character strengths to a professional development plan.	7.3 Reflects on personal actions and uses feedback to plan improvement 7.4 Engages in self-directed learning 7.6 Engages in career planning	7.5 Attends to wellbeing of self and others
DVM3 VPP3	Sustainable engagement / Burnout prevention	Intended learning outcomes Define sustainable engagement. Explain the factors that influence sustainable engagement, both positively and negatively. Demonstrate understanding of burnout. Progress expectations Evaluate the challenges and barriers to sustainable engagement. Apply an understanding of strategies and interventions that support sustainable engagement to create a personalized wellbeing and engagement plan.	7.2 Practices time management 7.3 Reflects on personal actions and uses feedback to plan improvement 7.5 Attends to wellbeing of self and others	7.4 Engages in self-directed learning 6.1 Solicits, respects, and integrates contributions from others 6.2 Functions as a leader or team member based on experience, skills, and context
DVM3 VPP4	Emotional intelligence	Intended learning outcomes Define emotional intelligence and explain its importance to veterinarians. Identify strategies to build emotional intelligence and to create emotional boundaries. Progress expectations Demonstrate the capacity to navigate emotional situations and to self-regulate emotional responses.	7.3 Reflects on personal actions and uses feedback to plan improvement 7.5 Attends to wellbeing of self and others	5.2 Adapts communication style to diverse audiences 6.1 Solicits, respects, and integrates contributions from others 6.2 Functions as a leader or team member based on experience, skills, and context

CBVE® = Competency-Based Veterinary Education. IVP = Introduction to the Veterinary Profession; VPP = Veterinary Professional Practice. Primary alignment indicates the competencies a topic most directly develops; secondary alignment indicates competencies it supports indirectly.

As Table 2 shows, the curriculum demonstrates a coherent developmental arc. DVM1 emphasizes foundations: wellbeing literacy, resilience, professional identity formation, diversity and inclusion, and career aspirations. DVM2 moves toward internal psychological resources, including mindfulness, self-compassion, and character strengths. DVM3 shifts toward professional sustainability through sustainable engagement, burnout prevention, and emotional intelligence. This sequencing is broadly aligned with the increasing demands students encounter as they move toward the clinical curriculum in the fourth year. The mapping revealed two important caveats. First, several outcomes imply applied developmental performance and are not yet consistently supported by practice, feedback, or assessment. For example, an outcome asking students to demonstrate capacity to navigate emotional situations and self-regulate emotional responses cannot be fully achieved through a single large-group seminar. Second, the curriculum articulates individual capabilities without making systems-level and relational contributors to wellbeing explicit.

### 3.2 Research question 2: appropriateness of teaching resources and session designs for achieving the intended learning outcomes

The curriculum demonstrates several important design strengths. It is embedded within credit-bearing coursework, uses a blended delivery approach, and includes small-group tutorials that support reflection, discussion, and professional application. Judged against the intended learning outcomes, however, the review identified an uneven fit between session format and outcome level. Learning outcomes designed at the level of defining, describing, and explaining are well supported by the current mix of online pre-work, seminars, and tutorials. Learning outcomes that imply applied developmental performance, such as demonstrating the capacity to navigate emotional situations and to self-regulate emotional responses, are supported by comparatively limited opportunities for practice, feedback, or observation. Facilitator resources varied in currency and in the amount of guidance offered to less experienced tutors, and the links between wellbeing concepts and professional practice, communication, and clinical learning contexts were more often left implicit than made explicit within session materials.

A second layer of analysis used WILD as an evaluative lens on the learning design of existing sessions. As noted in Section 3.4, the curriculum was not designed using WILD; the framework was applied here to describe the extent to which wellbeing-supportive design features are already present in current practice, and where they are not yet consistently visible. Evaluated in this way, the curriculum showed clear and consistent strengths in reflective practice, which is embedded in intended learning outcomes and progress expectations in all 3 years, and in the conditions for connection created by the small-group tutorial format. Other WILD elements were present, and uneven. Rationale and purpose were communicated in some sessions, and were not consistently framed in terms of the professional capability being developed. Transition-in and transition-out practices, explicit framing of psychological safety, and structured student choice appeared in individual sessions and appeared to depend substantially on the practices of individual educators rather than being designed features of the curriculum. Guidance on self-as-educator practices was not evident within the facilitator materials reviewed. Supplementary Table 1 summarizes this WILD-informed evaluation and the corresponding opportunities identified.

### 3.3 Research question 3: curriculum gaps and priorities for future development

Several curriculum development priorities were identified by the review team on the basis of the CBVE® mapping, the WILD-informed evaluation of learning design, and collaborative discussion between Melbourne Veterinary School educators responsible for the curriculum and wellbeing science experts external to it. The first was the need for a shared school and organization-level definition of wellbeing. Although wellbeing concepts are already present across the curriculum, the review identified an opportunity to make the underlying model and language more explicit and consistent. A working definition should be practical, context-sensitive, and co-designed with representative input from students, educators, clinical supervisors, professional staff, school leadership, and wellbeing and education partners. This definition could recognize wellbeing as both an individual capability and a property of learning and workplace environments. Establishing wellbeing as a shared core value would provide a clearer foundation for curriculum development, educator professional development, assessment, evaluation, and communication.

A second priority was to strengthen the third year of the curriculum, as a transition year between the pre-clinical and clinical curriculum. The review identified the third year as a point where students begin preparing more explicitly for the demands of clinical learning, professional responsibility, emotionally complex situations, feedback, uncertainty, and sustainable career development. Existing content on sustainable engagement and burnout prevention provides a strong foundation. This transition could be further strengthened through scenario-based practice, structured feedback, reflection, preparation for demanding professional scenarios, and job and life crafting activities that connect values, strengths, purpose, barriers, pathways, support systems, and sustainable career design.

A third priority was to extend wellbeing learning more intentionally into fourth-year clinical contexts. The identified gap was not the absence of mentoring, as Melbourne already has an existing staff mentor program in which students develop mentor relationships in DVM3 and continue into DVM4 unless they choose to change mentors. Rather, the gap was the limited explicit integration of wellbeing, professional identity formation, and portfolio-based reflection within existing clinical-year structures. Recommendations therefore focused on strengthening the wellbeing and professional development functions of current mentoring and feedback systems rather than creating a separate clinical-year wellbeing program. Low-burden options include brief group check-ins, asynchronous portfolio prompts, and the use of existing feedback review points as natural opportunities for wellbeing, professional identity formation, and growth-oriented conversations.

A fourth priority was to integrate wellbeing more explicitly with communication and clinical teaching. Communication education is a high-yield integration point because many wellbeing-relevant professional challenges are relational, emotionally charged, and communication-intensive. The Calgary-Cambridge Guide’s distinction among content, process, and perceptual skills helps clarify this connection (59). Perceptual skills draw attention to the clinician’s internal experience, including self-awareness, emotional regulation, assumptions, biases, mindfulness, reasoning, and reflection. These skills provide a natural bridge to wellbeing capacities that can be developed through practice, such as awareness, connection, insight, and purpose (23). In this way, communication teaching can support both observable communication behaviors and the wellbeing capacities that help students be successful in challenging interactions.

A final priority was to make relational and systems-level contributors to wellbeing more visible throughout the curriculum. The current curriculum includes strong attention to individual capabilities, and students may also benefit from more explicit opportunities to consider how learning environments, cultures, and systems shape wellbeing. This framing helps avoid positioning wellbeing as an individual burden and instead supports students to ask what they can shape personally, what requires support from others, and what requires collective or structural change.

Supplementary Table 2 illustrates how this integration could work in practice within existing communication teaching. The Calgary-Cambridge communication process skills are presented first (43), so that the professional capability being developed is explicit; the second column identifies evidence-informed wellbeing strategies that support the development of those skills, drawing on the trainable wellbeing capacities of awareness, connection, insight, and purpose described by Dahl et al. (23).

### 3.4 Research question 4: assessment tasks that could align with intended learning outcomes

At the time of review there were no explicit assessment tasks attached to the embedded wellbeing content. Students do complete reflective assessment tasks within the professional practice subjects, and some students choose to reflect on wellbeing-related competencies or experiences. Wellbeing-related learning is not yet itself assessed. This is the clearest opportunity for constructive alignment identified by the review. Constructive alignment requires that intended learning outcomes, learning activities, and assessment cohere (34), where wellbeing outcomes are taught but never assessed, that alignment is not present, and the content risks being read by students as peripheral to the professional competencies that matter at graduation (14). Assessment is also the mechanism through which developmental progress becomes visible. Without it, neither students nor educators can determine whether wellbeing-related professional capabilities are in fact developing across the program.

Many wellbeing-related learning outcomes imply developmental performance: students are asked to reflect, evaluate, formulate plans, apply strengths, navigate emotional situations, set boundaries, seek support, and connect wellbeing to professional practice. These outcomes require formative, applied, reflective, and longitudinal assessment rather than only knowledge assessment. A practical pathway forward is to use low-stakes completion-based tasks in the short term while making selected artifacts meaningful through portfolio pedagogy across the program. There is nuance in how to assign and assess reflective artifacts, so they do not become meaningless tick-box tasks. Ideally, students would return to selected artifacts over the course of the DVM curricular arc to demonstrate growth, insight, professional identity development, and professional application over time. Assessment can also be integrated into existing communication and clinical teaching. After a communication simulation, for example, students might reflect on both communication process and perceptual skill: what they noticed internally, how they regulated their internal response, what helped them remain present, and what they would like to try next time. This strengthens wellbeing and communication outcomes simultaneously and avoids creating a separate assessment stream.

Supplementary Table 3 describes an example developmental assessment sequence that would address this gap using structures already present in the program.

### 3.5 Research question 5: evaluating the impact of the curriculum on student learning and the learning environment

The review identified a need for a longitudinal evaluation strategy to examine both student learning and the learning environment over time. At present, the wellbeing curriculum includes relevant content, reflective learning activities, and opportunities for professional application. There is not yet a clearly articulated evaluation approach that follows implementation, student experience, capability development, clinical transfer, and learning environment outcomes across the DVM program. A key finding from the review was that evaluation should not focus only on whether students could recall wellbeing concepts. Because the curriculum aims to support professional capability development, evaluation should also examine whether students can apply wellbeing-related capacities and demonstrate relevant wellbeing competencies. Evaluation should further consider whether the learning environment itself reflects the principles being taught, including psychological safety, inclusion, autonomy support, connection, reflective practice, and growth-oriented feedback. Five evaluation foci were identified as necessary to capture this: implementation and feasibility, immediate student experience, developmental capability, clinical transfer, and learning environment alignment.

## 4 Discussion

### 4.1 Summary of principal findings

This review examined how wellbeing science is currently embedded within the Melbourne DVM curriculum, and how that integration might be strengthened within existing structures. In relation to the first research question, the existing wellbeing-related intended learning outcomes were appropriate for a multi-year, sequenced curriculum and conceptually well aligned with CBVE® 2.0, most strongly with Domain 7 and with secondary alignment across Domains 5 and 6. The curriculum follows a coherent developmental arc from foundational wellbeing literacy in DVM1, to internal psychological resources in DVM2, to professional sustainability in DVM3. Several outcomes imply applied developmental performance that current session formats cannot fully support, and the outcomes articulate individual capabilities without making systems-level and relational contributors to wellbeing explicit.

Teaching resources and session designs were mapped to knowledge-level and reflective outcomes but less well mapped to outcomes requiring applied practice and feedback. Evaluated through the WILD lens, wellbeing-supportive design features such as reflective practice and connection were clearly evident, while rationale-setting, transition practices, self-as- educator, psychological safety framing, and structured student choice were present unevenly and appeared to depend substantially on individual educators rather than on curriculum design. Five development priorities were identified: establishing a shared school-level definition of wellbeing; strengthening DVM3 as a transition year; extending wellbeing learning into DVM4 through existing mentoring and portfolio structures; integrating wellbeing with communication and clinical teaching; and making relational and systems-level contributors to wellbeing visible across the curriculum. The absence of explicit assessment of wellbeing learning emerged as the clearest point of incomplete constructive alignment, with a developmental, portfolio-anchored assessment sequence identified as a feasible response. The curriculum did not yet have an articulated evaluation strategy spanning implementation, student experience, capability development, clinical transfer, and learning environment outcomes.

### 4.2 Interpreting the findings through wellbeing science and competency frameworks

The pattern identified here, of conceptually sound wellbeing content that is not yet fully embedded within assessment, learning design, and the learning environment, is consistent with what has been described in higher education more broadly. Lister and Allman (14) propose that wellbeing is embedded in a curriculum when it is modeled in pedagogical practice, tailored to student needs, inherent in institutional culture, sustained through partnership, and represented in strategy with appropriate resourcing. Measured against those criteria, the curriculum reviewed here is well developed with respect to content and reflective practice, and is still developing with respect to pedagogy, assessment, culture, and strategy. This is not a weakness specific to this program. It reflects the broader state of the field of veterinary education, in which wellbeing content has typically been introduced into professional curricula ahead of the pedagogical, assessment, and governance structures needed to sustain it (39).

The findings also align with veterinary-specific scholarship arguing for a both/and approach that builds individual capabilities while addressing structural and cultural contributors to distress (12, 32, 44). The observation that wellbeing-supportive design features depend substantially on individual educators is consistent with SDT-informed evidence that the social climate of a learning context predicts psychological need satisfaction and frustration and, through these, hope, life satisfaction, and burnout among veterinary students (29, 31). If the learning environment is a determinant of student wellbeing, then variability in educator practice is not a peripheral implementation detail, it is a substantive and crucial element of the conditions under which the curriculum and educational program is experienced. Although SDT provides an important account of how learning environments support or frustrate autonomy, competence, and relatedness, it does not exhaust the relational mechanisms relevant to wellbeing-integrated education. Social interdependence theory proposes that the ways in which learning goals are structured shape interaction among learners: positive interdependence can promote cooperation, interpersonal support, perspective-taking, and shared responsibility (45).

This work is situated within broader One Health and global education priorities by recognizing that veterinary professional wellbeing is linked to the quality and sustainability of animal care, community health, public service, and veterinary systems (11). The Sustainable Development Goals (SDGs) provide the broader professional and societal rationale for the work; wellbeing science explains why learning environments matter for human flourishing; WILD offers a practical, theory-informed approach for embedding wellbeing into curriculum design; and CBVE® provides a structure for aligning wellbeing-related capabilities with professional competencies. Within this broader frame, the findings should be interpreted as identifying an upstream contribution rather than demonstrating a direct effect on One Health or SDG outcomes. The review did not evaluate animal, public, environmental, or planetary health outcomes. Instead, it identified curriculum structures that may help students develop the professional capabilities and sustainable engagement needed to contribute to these interconnected responsibilities over time.

A further observation concerns how the opportunities described in the next section were generated. The development priorities reported in the results section emerged directly from the curriculum review process. The opportunities described below emerged instead from the wellbeing science literature, and were surfaced through sustained collaboration between veterinary educators and wellbeing science experts. This distinction matters practically. It suggests that partnership between veterinary educators and wellbeing scientists is itself a mechanism for curriculum evolution: it gives veterinary programs access to evidence-based practices they are unlikely to encounter through discipline-specific literature alone, and it gives wellbeing scientists the professional and curricular context needed to make those practices feasible, proportionate, and relevant. The recommendations that follow are therefore offered not only as content, but as an illustration of what such partnerships can produce.

### 4.3 Practical recommendations for strengthening embedded wellbeing curricula

#### 4.3.1 Strengthening learning design and teaching practice using the WILD framework

Findings related to Research Question 2 indicated that wellbeing-supportive learning design features were present but inconsistently specified in teaching and facilitator materials and often depended on the practices of individual educators. This finding identifies a feasible opportunity to strengthen constructive alignment without adding substantial content or workload. Although constraints such as room allocation, class size, contact time, and scheduling may be difficult to change, educators can deliberately shape the immediate learning environment through small, intentional design choices. These include structuring how students enter and leave a session; communicating its relevance and purpose; normalizing mistakes and productive failure; wording prompts to support reflection and participation; explicitly establishing psychological safety; offering bounded opportunities for student choice; and using guided reflection to connect wellbeing concepts with professional practice. Within the WILD framework, these practices are mechanisms for embedding wellbeing into teaching and learning rather than supplementary teaching techniques (16, 33). Incorporating selected practices consistently into session plans and facilitator guides could reduce reliance on individual educator discretion and support more consistent implementation across the DVM program. Supplementary Table 4 provides practical examples aligned with each WILD learning design element.

#### 4.3.2 Additional wellbeing science opportunities

Several additional opportunities emerge from the wellbeing science literature. These are not separate content areas to be added to an already-full curriculum; they are wellbeing science interventions and theories that could be woven through existing sessions, reflective assessments, coaching and mentoring conversations, and any future curricular integration.

Job crafting and life crafting interventions may be especially useful as students prepare for clinical placements and transition to practice. Job crafting refers to the ways individuals intentionally shape aspects of their work, relationships, tasks, and mindset to increase meaning, engagement, and fit within their role, and has been associated with increases in job resources and wellbeing among working adults (46, 47). Life crafting similarly involves reflecting on values, strengths, goals, barriers, pathways, and supports in order to intentionally design a meaningful and sustainable life; crafting of both kinds has been linked with psychological need satisfaction, and life crafting interventions have been shown to support emerging adults in creating an authentic and meaningful life (48). Introduced early in professional identity formation and revisited before clinical rotations and again near graduation, these activities could help students clarify values, envision sustainable careers, and prepare to seek out, negotiate, and help co-create healthier and more meaningful work environments. These practices also move the curriculum beyond individual self-management toward an agentic, design-oriented stance on how students shape the work environments they will eventually inhabit (46, 47).

Wellbeing literacy is the capability to understand and intentionally compose wellbeing-related language and actions across contexts (25, 26). Wellbeing literacy could serve as a connective thread across the wellbeing curriculum, the communication curriculum, and professional identity formation. Making this capability explicit in the curriculum could help students and educators develop a shared language for discussing their own experiences, interpreting team and learning environments, and engaging more productively in feedback, peer support, and help-seeking. Wellbeing literacy also aligns closely with the CBVE® model’s emphasis on observable, assessable professional competencies, and offers a natural anchor for the longitudinal portfolio, mentoring and coaching conversations, and integrated communication and wellbeing assessments proposed elsewhere in this review.

Hope theory provides a practical framework for structuring goal-oriented conversations, reflective assessments, and coaching practices, particularly across the preclinical to clinical year transition, when students are asked to envision themselves as future veterinary practitioners amid significant uncertainty (49). Traditionally conceptualized as the interaction of agency (WillPower) and pathways thinking (WayPower), hope theory has been recently reconceptualized from a dynamic systems-informed approach to include collective dimensions such as WePower (shared goal pursuit) and WhyPower (purpose and meaning) (50). Hope as a systems thinking skill is associated with intrinsic motivation, an orientation toward mastery rather than toward goals focused on one’s performance relative to others, resilience in the face of adversity, and stronger academic outcomes (50). Integrating hope theory into reflective prompts, coaching conversations, and peer learning circles could strengthen both intrinsic motivation and wellbeing, while building on the learning outcomes already established across DVM1 to DVM3.

#### 4.3.3 Educator capability and educator wellbeing

Embedding wellbeing across the curriculum also requires attention to educator capability and educator wellbeing. The hidden curriculum is well established in veterinary education, and faculty modeling, feedback practices, and relational presence may be among the highest-leverage points for sustainable change (51–53). Needs-supportive teaching, which includes clear rationales, meaningful choice, developmental feedback, and warm relational presence, has been positively associated with student wellbeing across cultural contexts in school settings (28, 54). It is best understood as an ongoing teaching practice rather than as a discrete intervention: some educators are naturally need-supportive without any formal program, and the developmental task for a school is to make these behaviors explicit, shared, recognized, and sustainable across a teaching team. Investment in veterinary professional educator wellbeing functions simultaneously as a wellbeing intervention and as a mechanism for higher-quality learning, and it complements the WILD framework by attending explicitly to the self-as-educator and to the learning climate as a determinant of wellbeing, not only to the content delivered within it (16, 33).

#### 4.3.4 Making relational and systems-level contributors to wellbeing visible

The current curriculum includes strong attention to individual wellbeing capabilities. A next step is to make relational and systems-level contributors to wellbeing more explicit across existing topics, without adding new sessions. For example, mindfulness can be paired with the question, “What conditions make it easier or harder to be present?” Self-compassion can be paired with, “How do feedback norms influence growth?” Emotional intelligence can be paired with, “How do team cultures and leadership practices shape whether people feel safe to speak up?” Job and life crafting can be paired with, “What can I shape individually, what requires support from others, and what requires leadership or structural change?” These paired questions help students understand wellbeing as a *shared responsibility* shaped by individuals, relationships, learning environments, and professional systems, and they operationalize the both/and approach advocated in the contemporary veterinary wellbeing literature (12).

#### 4.3.5 Evaluating impact over time

The review supports a staged, mixed-methods evaluation approach that begins with implementation and feasibility, then moves toward immediate student experience, developmental capability, clinical transfer, and learning environment indicators. Implementation and student experience data can be collected quickly and at low cost, and can be used to refine sessions before more resource-intensive longitudinal capability and clinical transfer evaluation is attempted. Supplementary Table 5 describes an example evaluation strategy.

### 4.4 A proposed ecological framework for embedding wellbeing in veterinary education

The findings in this review support an emerging ecological and systems-based theoretical framework for embedding wellbeing science in veterinary education. The proposed framework positions wellbeing as an emergent property of interacting individual and systems conditions, and encompasses four interacting components: (1) individual wellbeing capability, comprising wellbeing literacy and the learnable psychological capacities through which it is enacted; (2) evidence-informed and ethically grounded program, learning environment, and assessment design; (3) governance structures and leadership commitment that sustain implementation; and (4) a shared consensus model that aligns educational practice with flourishing as a core aim. Individual wellbeing capability provides the foundation: wellbeing literacy enables students to recognize, understand, and communicate about wellbeing, while the capacities of awareness, connection, insight, and purpose represent the teachable psychological skills through which that literacy is enacted in practice. Relational and organizational constructs such as emotional intelligence and psychological safety highlight the importance of interpersonal and cultural dynamics in shaping wellbeing outcomes. Systems-informed curriculum design ensures that these capabilities are supported through pedagogy, assessment, and institutional culture rather than isolated programming. Industry and policy-level lenses further emphasize that sustainable change requires coordinated action across the educational pipeline, workplace environments, and professional systems. This proposed framework reframes wellbeing from an add-on initiative to a systemic feature of educational ecosystems (13). It should be understood as a preliminary synthesis generated from the current curriculum review and supporting literature, rather than a fully developed or empirically tested model; further conceptual elaboration, feasibility testing, and evaluation across diverse veterinary education contexts will be needed in future studies. Table 3 summarizes the proposed framework.

**Table 3:** Proposed ecological framework for embedding wellbeing in veterinary education.

Ecological component	Key question	How the literature contributes	Application to veterinary education
1. Individual wellbeing capability (wellbeing literacy and learnable wellbeing capacities)	What capabilities allow students to understand, communicate about, and enact wellbeing?	Wellbeing literacy is defined as the capability to comprehend and intentionally compose wellbeing language and actions within context (25, 26). Wellbeing science further proposes that flourishing is supported by four trainable capacities: awareness, connection, insight, and purpose, cultivated through intentional mental training and reflective practices (23). Humanities-informed approaches highlight meaning-making, narrative, and identity exploration as central to purpose and motivation (55). Together, these framings position wellbeing as teachable, learnable, innate capacities to be strengthened, rather than a coping strategy.	Positions wellbeing as an explicit professional competency aligned with CBVE®, supporting reflective practice, self-awareness, and communication about wellbeing within professional contexts.
2. Program, learning environment, and assessment design (evidence-informed, ethical, theory-driven integration)	How should wellbeing be integrated into veterinary curriculum, learning design, and assessment?	The embeddedness framework suggests wellbeing is integrated when it is modeled in pedagogy, tailored to student needs, supported culturally, sustained through partnerships, and represented in institutional strategy (14). Wellbeing Integrated Learning Design (WILD) emphasizes embedding wellbeing within learning design rather than isolated programs (16, 33)	Supports vertical integration and encourages alignment between intended learning outcomes, andragogy, and assessment.
3. Governance (institutional leadership, accreditation, and structural alignment)	What institutional and systemic conditions sustain wellbeing integration?	The World Health Organization (WHO) Wellbeing Framework emphasizes governance, cross-sector coordination, and structural accountability for wellbeing outcomes (56). Systems-informed wellbeing science and education literature supports that institutional leadership, faculty development, and policy alignment are necessary to sustain wellbeing initiatives (57, 58)	Aligns wellbeing curriculum implementation with institutional strategy, accreditation expectations, and faculty development initiatives that support teaching and assessment practices.
4. Consensus model of flourishing (shared philosophical foundation for the aims of education)	Why should education prioritize flourishing as a core aim?	A consensus model of wellbeing in education argues that flourishing provides an ethically defensible and empirically supported aim for educational systems (13). Needs- supportive teaching elevates both wellbeing and academic success (54).	Provides philosophical grounding for integrating wellbeing within CBVE® and accreditation processes, positioning student flourishing as both a core day-one competency and a mechanism through which effective teaching and learning occur.

## 5 Implications for veterinary education and the veterinary workforce

This evaluative review has implications beyond a single institution. Veterinary education is a contextual setting and a system where future professionals can experience and learn how to manage occupational demands and stresses as individuals, as well as how to contribute to and lead within healthier teams, cultures, and workplaces. Integrating wellbeing science with CBVE® makes wellbeing visible as a professional capability: skills that learners can practice, receive feedback on, and develop over time. The integration of wellbeing and communication may be especially important for workforce and career sustainability. Many occupational stressors in veterinary practice are relational and communication-intensive: challenging client interactions, end-of-life conversations, conflict, feedback, teamwork, dealing with uncertainty, ethical challenges, and moral distress. Teaching communication skills without attention to the learner’s internal state risks underpreparing students for the emotional labor of real-life practice and professional contexts. Conversely, teaching wellbeing without application to communication and real-life contexts risks leaving wellbeing as abstract or peripheral content.

This review also underscores the need to include the broader veterinary healthcare team in future work. Although the review focused on a doctor of veterinary medicine curriculum as the case example, the same systems-informed approach can apply to the education of veterinary nurses, veterinary technicians and technologists, veterinary assistants, veterinary practice managers, veterinary social workers, veterinary leaders and educators, and support staff. A flourishing veterinary workforce will require interventions that operate at individual, team, organizational, educational, and policy levels, with attention to achieving harmony among the levels and impact over time. Veterinary education can contribute to sustainable wellbeing and flourishing in the profession by preparing graduates to be the positive change makers that help enable individuals and systems to support sustainable careers, thriving workplaces, and high-quality patient care.

## 6 Strengths, limitations, and future directions

This review has a number of strengths. It examines a functioning, credit-bearing, longitudinal wellbeing curriculum rather than a discrete or short-term intervention, addressing a recognized gap in the veterinary education literature. It brings together CBVE® and the emerging innovative wellbeing-informed learning design framework, and applies them to the same curriculum. The review team combined insider knowledge of the curriculum and institutional context with perspectives from veterinary education and wellbeing science experts who had not participated in the original curriculum’s design or delivery, supporting contextual accuracy alongside critical examination. The recommendations were developed with explicit attention to feasibility within real constraints of timetabling, clinical workload, educator capacity, and assessment burden, and are therefore intended to be practical. Finally, the evaluative review process itself is replicable: the mapping approach, the evaluative lenses, and the guiding questions can be applied by other veterinary programs to their own curricula, and the resulting artifacts can be adapted as working tools.

Several limitations should also be acknowledged. This is a theory-informed evaluative case study of one veterinary program and is not intended to establish generalizable effectiveness. The review relied primarily on curriculum documents, mapping, observations, and collaborative review and evaluation with wellbeing science experts. The unit of analysis was the formal curriculum rather than the curriculum as experienced by students and educators. Systematic student and educator feedback was not collected; consequently, the review cannot determine whether the curriculum was perceived as relevant, psychologically safe, useful, or feasible, nor whether intended learning outcomes were achieved in practice. The evaluative interpretations and tables should therefore be understood as theory-informed analyses of curriculum design rather than evidence of learner experience or program effectiveness. A subsequent mixed-methods and participatory evaluation should examine how the curriculum is enacted and experienced, including perspectives from students across DVM1–DVM4, educators, clinical supervisors, and recent graduates.

The evaluative observations reported here reflect the judgements of the review team applying external frameworks to existing materials and observed sessions, and a different team might weight these judgements differently. The authors’ relationships to the curriculum should also be considered when interpreting the findings. LD had contributed to its implementation and delivery, and LD and JC held roles involving curriculum oversight and contextual knowledge of the DVM program. These perspectives strengthened the contextual accuracy and feasibility of the review but may also have influenced how evidence and priorities were interpreted. This influence was addressed through primary analysis by VKC, explicit use of external analytic frameworks, iterative review by authors external to the curriculum, and consensus discussion; however, these processes do not eliminate interpretive subjectivity.

These limitations point toward the future work most needed in this area. Priorities include evaluating the implementation of WILD-informed learning design changes and their effect on student experience and psychological need satisfaction; testing whether developmental, portfolio-anchored assessment of wellbeing capabilities is feasible and educationally relevant and valuable within competency-based programs; examining whether wellbeing learning transfers into clinical years and early practice; incorporating student and graduate perspectives directly into curriculum review; and applying the same review process across multiple veterinary schools and to the education of the wider veterinary team, to establish whether the patterns identified here are program-specific or characteristic of the field.

## 7 Conclusion

The Melbourne DVM wellbeing curriculum represents an opportune case example for what embedding wellbeing science can look like within contemporary veterinary education. The opportunity illuminated by this review is to focus less on adding new topics and more on strengthening embeddedness, application, and integrated assessment and evaluation. In this case example, the highest-leverage starting points are investing in educator capability and wellbeing, and aligning assessment with the wellbeing outcomes that are already being taught. A formal outcomes assessment evaluation will refine and enhance this review process and inform a planned curricular revision process. For the broader veterinary profession, this review illustrates how educational systems can contribute to workforce wellbeing from an upstream approach. A paradigm shift towards teaching *for* wellbeing instead of teaching *about* wellbeing in veterinary professional programs can create systemic change, wider ripple effects, and positive impacts for the veterinary professional workforce.

## Data Availability

The original contributions presented in the study are included in the article/Supplementary material, further inquiries can be directed to the corresponding author/s.
