# Supplementary material for: An upstream approach to veterinary workforce wellbeing: an evaluative review of an embedded wellbeing curriculum in veterinary education
Source: Front Vet Sci. 2026 Sep 15;13:1972718. doi: 10.3389/fvets.2026.1972718 (PMC13619591; doi:10.3389/fvets.2026.1972718)
Supplement: Supplementary file 1 [file Data_Sheet_1.PDF]

Supplementary Material

Supplementary Table 1. WILD-informed evaluation of the current wellbeing curriculum teaching resources and learning design

**WILD learning design element**	**Observation from the curriculum review**	**Opportunity identified**
Rationale and purpose	Sessions introduce topic content clearly. The rationale for why the topic matters to professional practice, and how it builds professional capability, is made explicit in some sessions and is not a consistent feature of session materials.	State the purpose of each session in terms of the professional capability being developed and its link to CBVE competencies.
Transition-in practice	Sessions typically open with content delivery. Structured grounding or check-in practices at the start of sessions were not documented in the facilitator resources reviewed.	Add a brief grounding or check-in practice at the start of each session, modelled by the educator.
Connection and psychologically safe learning environment	Small-group tutorials create conditions for connection and discussion, and represent a clear strength. Explicit co-creation of learning environment norms, and explicit framing of psychological safety, were not evident in the reviewed materials.	Co-create learning community norms in the first session of each subject, and name psychological safety explicitly as a condition for reflective discussion.
Teaching style	Delivery is predominantly educator-directed, with tutorials providing opportunities for discussion. Opportunities for student-	Sequence activities from low-stakes paired discussion toward small-group role play and

**WILD learning design element**	**Observation from the curriculum review**	**Opportunity identified**
	directed and self-regulated learning are present, and limited.	simulation, with reflection and feedback after each step.
Student choice and flexibility	Reflection prompts and activity formats are generally prescribed. Structured choice was not a consistent design feature across sessions.	Offer a small number of alternative reflection prompts, and choice of response modality, where appropriate.
Micro-moments of wellbeing	Positive emotion, movement, and shared voice appear to be generated informally by individual educators rather than designed into session plans.	Build brief, structured activities that generate connection and positive emotion into session plans, so that they do not depend on individual educator practice.
Reflective practice	Reflection is a consistent strength. It is embedded in intended learning outcomes and progress expectations across all three years and is supported by the tutorial format.	Extend reflection from within-session activity to longitudinal, portfolio-anchored reflection that students revisit across the program (see Section 4.4).
Transition-out practice	Sessions generally close with a content summary. Structured closing practices such as exit tickets or commitment-to-action prompts were not documented in the materials reviewed.	Close sessions with a brief exit ticket capturing one insight, one action, and one support need.
Self-as-educator	Facilitator resources focus on content and activity instructions. Guidance on educator wellbeing, presence, and self-as-educator practices was not evident.	Include self-as-educator guidance within facilitator resources and address it directly in educator professional development (see Section 5.3.3).

WILD = Wellbeing Integrated Learning Design Framework (Colla and Mossman 2023; Mossman and Colla 2026)

Supplementary Table 2. Integrating evidence-informed wellbeing strategies into Calgary-Cambridge communication process skills teaching

Calgary-Cambridge communication process skills	Evidence-informed wellbeing strategies that support development	Example integration
Initiating the session and providing structure: preparation and focusing attention; identifying the problem list; negotiating the agenda; providing structure through summaries, signposting, logical sequencing, and timing; shared planning and contracting next steps.	Grounding and awareness practices; coaching-informed questions; job and life crafting. Supports awareness and purpose.	Use pre-brief grounding before communication simulations, then ask students to identify their goal for the encounter, how they maintained focus, and one communication choice that supported the shared agenda.
Gathering information and responding to cues: listening without interruption; facilitation and attentive listening; picking up verbal and non-verbal cues; accepting legitimacy without judgment; empathy and acknowledgement; responding to client cues and eliciting reactions or feelings.	High-quality connections; active constructive responding; deep listening; clinical debrief; experiential learning. Supports awareness, connection, and insight.	During difficult-client or emotionally charged conversations, pause the debrief around one cue the student noticed, what they inferred, how they regulated their response, and how they adapted their next question or empathic statement.
Explanation and planning: demonstrating confidence; using notes without interfering with the	Experiential learning; reflective practice; coaching-informed	Invite students to design a client-friendly explanation, visual aid, or teach-back strategy that reflects their own

**Calgary-Cambridge communication process skills**	**Evidence-informed wellbeing strategies that support development**	**Example integration**
relationship; sharing thinking and explaining rationale; assessing the client’s starting point; asking what else would be helpful; visually conveying information or writing information down.	questions; teach-back. Supports awareness, insight, and purpose.	communication style while remaining client-centered. Feedback can ask: What did you create? What client need did it serve? What would you adapt next time?
Building the relationship: greeting and demonstrating respect; understanding the client’s ideas, concerns, expectations, and effects on life; encouraging feelings; providing support; dealing sensitively; involving the client and encouraging client input.	High-quality connections; active constructive responding; perspective-taking; deep listening. Supports connection and purpose.	In empathy, reflective listening, or success-sharing practice, have students practice active constructive responding, perspective-taking, and supportive statements. Feedback can include one observed behavior that strengthened trust, belonging, or shared understanding.
Presence and non-verbal behavior: putting aside the last task and focusing attention; appropriate non-verbal behavior; attending to timing and task flow; chunking and checking.	Grounding and awareness practices; the clinical or medical pause; brief breathwork or body scan practices. Supports awareness.	Begin communication labs with a 60-second transition-in practice such as grounding, breath awareness, or body scan. After the encounter, ask students to note whether presence changed their listening, non-verbal behavior, or ability to chunk and check information.
Boundaries, closure, and safety-netting: negotiating the agenda; attending to timing and task boundaries; sharing options rather than directives; safety-	Job crafting and life crafting; clinical debrief; reflective practice; coaching-informed questions. Supports	Use role-play scenarios involving competing demands, time pressure, or emotional load. Students practice agenda-setting, boundaries, shared decision-making, and safety-netting. Feedback can

Calgary-Cambridge communication process skills	Evidence-informed wellbeing strategies that support development	Example integration
netting; contracting next steps; summarizing and checking agreement.	awareness, insight, and purpose.	ask students to identify what they could shape individually, what required team support, and what was a systems issue.
Perceptual skills across the consultation: all process skills, with emphasis on making organization overt, building relationships, incorporating the client's perspective, shared decision-making, and forward planning.	Clinical debrief; experiential learning; high-quality connections; job and life crafting; coaching-informed reflective questions. Supports awareness, connection, insight, and purpose.	Use a Calgary-Cambridge perceptual skills reflection after simulated or observed consultations. Students identify thoughts, feelings, assumptions, social cues, power dynamics, and environmental factors that shaped the encounter, then write one action to support wellbeing for self, client, patient, or team.

Communication process skills are drawn from the Calgary-Cambridge Guide (Kurtz and Silverman 1996; Shaw and Coe 2024). The wellbeing capacities referenced (awareness, connection, insight, purpose) are described in Dahl et al. (2020).

Supplementary Table 3. Example developmental assessment sequence

Timing	Assessment focus	Example task	Portfolio/feedback use
DVM1	Professional identity and wellbeing plan	Students identify values, strengths, supports, and one realistic wellbeing practice.	Portfolio reflection; exit ticket.
DVM2	Character strengths and self-compassion application	Students describe how they applied one strength or self-compassion strategy in a learning challenge.	Completion plus evidence of

Timing	Assessment focus	Example task	Portfolio/feedback use
			engagement; later revisit.
DVM3	Scenario-based emotional intelligence and sustainable engagement reflection	Students respond to an emotionally complex scenario and identify regulation, communication, support-seeking, boundaries, and systems factors.	Brief rubric assessing specificity, professional relevance, and next action.
DVM4	Clinical-year portfolio synthesis	Students select two prior artifacts and one clinical experience to describe how professional identity, communication, and sustainable career thinking have evolved.	Portfolio synthesis; mentor conversation guide.
Capstone / pre-graduation	Sustainable career design statement	Students articulate what they will seek, shape, negotiate, and contribute to in their first workplace.	Graduate transition statement; employment interview preparation.

Supplementary Table 4. WILD-informed learning design elements and practical examples for strengthening teaching resources and learning activities

WILD framework learning design element	Practical examples
Identifying a rationale/purpose	Introduce the session with a link to the literature and to the purpose of how the learning outcomes build wellbeing capabilities and professional competencies.
Transition-in practice	Begin each session with a brief grounding and check-in practice.<br><br>Examples:<br><br>Grounding (pre-class): educator practice before coming into the teaching space.<br><br>Grounding (start of class): educator-led practice.<br><br>Mindful breathing (box breathing, 4-7-8 breathing), poem, meditation, chair yoga, humor, bringing forth a character strength.<br><br>Check-in practice: create check-in groups of 3-4 students, and provide a choice of prompts or questions to discuss for a few minutes before starting class.
Create connection and a safe environment	Co-create the learning environment: the educator leads a group formation activity in the first session, and students co-design their learning community and learning environment in the subject.<br><br>Examples:<br><br>Learning community: invite students to introduce themselves to help build up a picture of the class community (for example, using Padlet); create a survey to gauge student interests and share results with the class.<br><br>Icebreaker activities: Wall of Wonder. A WILD-informed transition-in activity in which students write a question they would like to be asked by peers and place it on a shared "Wall of Wonder." Students then select a question written by someone else and use it as a prompt for small-group conversation. This activity

**WILD framework learning design element**	**Practical examples**
	supports student choice and flexibility, creates connection, and can generate positive emotions at the start of a session.
Teaching style	Heutagogy: create the structure for self-determined learning and then respond to student direction.<br><br>Examples:<br><br>Student-led questions, with the teacher responding to students' reflections and questions.<br><br>Sequence from low-stakes think/pair/share practice to small-group role play to simulation, with reflection and feedback after each step.
Student choice and flexibility	Examples:<br><br>Offer two or three reflection prompts for students to choose from.<br><br>Offer choice in modality in how students respond; for example, students choose written, verbal, or brief portfolio or reflection formats when appropriate.
Creating micro-moments of wellbeing	Examples:<br><br>Structured think/pair/share activities.<br><br>Positive emotions: "Snowball activity", a brief, energizing opening practice designed to create micro-moments of positive emotion, connection, movement, and shared voice at the start of class. Students respond to a simple reflection prompt on a piece of paper, crumple it into a "snowball," and gently toss it across the room. Each student picks up a snowball, reads the anonymous response, and shares it aloud or discusses it with a partner or small group.

WILD framework learning design element	Practical examples
Reflective practice	Build in reflective practice throughout the session, including ideas from Small Teaching (Lang, 2021), which relies on micro-interventions and quick modifications rather than major course overhauls.<br><br>Examples of quick, high-impact strategies are:<br><br>The first five minutes: opening poll (start by asking students what they already know about a subject to strengthen memory and activate prior knowledge); memory retrieval (begin by asking students to write down the three most important points from the previous session); prediction (present a new, complex problem and have students guess the outcome before you teach the underlying theory).<br><br>In-class engagement: one-minute paper (pause your lecture to give students exactly 60 seconds to summarize the concept you just covered in their own words); think-pair-share (pose a question, let students think individually, have them discuss with a neighbor, and then share with the class); jigsaw method (break a larger topic into small chunks, assign different groups to master one chunk, and have them teach it to the rest of the class).<br><br>The last five minutes: exit ticket (give students an index card or digital prompt); self-correction (ask students to reflect on an early misconception they held at the start of class and how it changed).
Transition out	Examples:<br><br>End the session with an exit ticket: one insight, one next action, one support needed.<br><br>Example exit ticket questions: What is one idea I am taking away? What is one small thing I will try? What would help me apply this? What is the most important thing you learned today, and why? How would you describe today’s

WILD framework learning design element	Practical examples
	lesson to a student who was absent? What is one lingering question you still have about today's material?<br><br>Small group circle up: reflect on the course as a group and invite students to share their takeaways in a safe space.<br><br>Legacy boards: in the final session of the subject, include an online legacy board created by students using images and responses to questions such as "what is one takeaway from this subject" and "what message would you like to share with next year's cohort?" These legacy boards are embedded in the homepage on the LMS of the next year's cohort so that when they join the subject they are welcomed by messages from former students and by what they can look forward to in the course.

Supplementary Table 5. Example integrated evaluation strategy for the embedded wellbeing curriculum

Evaluation focus	What the review identified	Guiding evaluation question	Possible data sources
Implementation and feasibility	The curriculum would benefit from clearer information about how consistently sessions are delivered and what support educators need.	Were sessions delivered as intended, and were the materials feasible and usable for educators?	Facilitator debriefs; resource audits; delivery notes; brief educator surveys; teaching team reflections.

Evaluation focus	What the review identified	Guiding evaluation question	Possible data sources
Immediate student experience	Existing sessions include relevant wellbeing content, but student perceptions of relevance, clarity, usefulness, and psychological safety should be evaluated more systematically.	Did students experience the sessions as relevant, clear, useful, and safe enough for participation and reflection?	Exit tickets; brief post-session surveys; qualitative comments; in-class reflection prompts.
Developmental capability	Many intended learning outcomes imply growth in reflection, self-awareness, communication, feedback engagement, help-seeking, and sustainable career thinking.	Can students demonstrate development of wellbeing-related professional capabilities over time?	Portfolio artifacts; scenario-based reflections; communication lab reflections; mentor conversation summaries; selected reflective assignments.
Clinical transfer	Wellbeing learning should become visible in clinical and professional contexts, particularly during DVM3-DVM4 transitions.	Do students apply wellbeing-related capabilities in clinical placements, teamwork, client communication, feedback, and emotionally complex situations?	DVM4 reflection prompts; clinical debriefs; placement feedback; mentor conversations; graduate follow-up.
Learning environment alignment	The learning environment may either reinforce or undermine the wellbeing	Do students and educators experience the learning environment as need-supportive, psychologically safe,	WILD-informed observations; focus groups; educator learning circles; psychological safety measures; student

**Evaluation focus**	**What the review identified**	**Guiding evaluation question**	**Possible data sources**
	principles taught in the formal curriculum.	inclusive, and aligned with wellbeing science principles?	and educator wellbeing or learning climate surveys.
